# Hémangiome vertébrale géant révélé par un traumatisme lombaire: à propos d'un cas

**DOI:** 10.11604/pamj.2014.18.351.2470

**Published:** 2014-08-29

**Authors:** Abdelilah Mouhsine, Adil Essanhaji, El Mehdi Atmane, Radouane Rokhssi, B Ouchaib Kadiri, Mbark Mahfoudi, Abdelghani El Fikri

**Affiliations:** 1 Service de Radiologie HMA, Marrakech, Maroc; 2 Service de neurochirurgie HMA, Marrakech, Maroc

**Keywords:** Hémangiome vertébrale, compression médullaire, traumatisme lombaire, vertebral hemangioma, spinal cord compression, lumbar trauma

## Abstract

L'hémangiome vertebral est une tumeur bénigne fréquente, c'est une lésion bénigne habituellement asymptomatique. Les formes évolutives résponsables de compression médullaire sont beaucoup plus rares, une surveillance clinique et radiologique est conseillé. Nous mettons en exergue à travers ce cas revelé par des signes de compression médullaire suite à une chute, l'intérêt de l'imagerie en coupes dans le diagnostic positif; pour déceler les formes compliquées, et pour orienter l'attitude thérapeutique. Les formes neurologiques nécessitent une prise en charge neurochirurgicale.

## Introduction

L'hémangiome vertebral est une tumeur bénigne relativement fréquente, d'origine malformative, constituée de vaisseaux sanguins néoformés de structure normale, sans shunt artério-veineux. Cette lésion bénigne demeure asymptomatique. Les formes évolutives résponsables de compression médullaire sont beaucoup plus rares, elles relévent d’ une prise en charge neurochirurgicale.

## Patient et observation

Patient de 54 ans avec ATCDs d'HTA suivie, présente suite a une chute un traumatisme lombaire. L'examen Clinique trouve un syndrome rachidien lombaire avec paraparésie cotée 2/5, un niveau sensitif ombilical, ROT abolish, des troubles sphincteriens (rétention urinaire). Le patient était exploré par une TDM suivie d'IRM lombaire, montraient une Fracture tassement du corps vertebral L5 avec recul du mur postérieur sur une lesion préexistante (hémangiome geant). Le patient a bénefécié d'une laminectomie decompressive suivie d'une radiothérapie complémentaire avec une dose totale de 35 grays. L’évolution Clinique était bonne.

## Discussion

L'hémangiome vertebral est fréquent (10 pour cent des autopsies systématique) [1] et represente la fréquente des tumeurs bénignes vertebrales. Il est géneralement asymptomatique et il est découvert a l'occasion d'un examen réalisé à titre systématique ou suite a un traumatisme commme dans notre cas. Il se voit chez l'adulte jeune avec une discréte prédominance féminine, 2 femmes pour un homme [2]. La localisation dorsale est la plus fréquente de D3 à D9 [2]. La localisation lombaire dont nos rapportons un cas a été rarement décrite dans la littérature [3].

**Figure 1:**
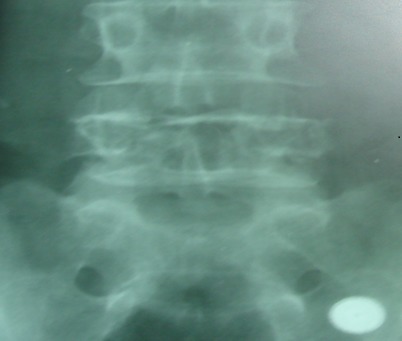
Rx standars lombosacré face: fracture tassement de L5

La symtomatologie clinique est le plus souvent représentée par une compression médullaire tel le cas de notre patient. Elle se manifeste plus rarement par des dorsalgies ou des radiculalgies [4]. La physiopathologie des signes neurologiques fait intervenir plusieurs paramétres. On géneral, il s'agit d'une extension épidurale, par contre les tassements vertebraux ou les hématomes compressifs n'intervient que rarement [4]. L'hémangiome vertebral agressif a un aspect typique sur les radiographies standards [5]. Il siége sur le corps vertébral qui est déminéralisé présentant des travées verticales réalisant l'aspect grillage caractéristique (Figure 1). L’étude TDM, lorsque l'aspect n'est pas typique, permet de préciser l’étendue de l'atteinte osseuse (pédicules, arc post) l'extension épidurale, l'hypervascularisation et le stroma tissulaire de la lésion [4].

**Figure 2:**
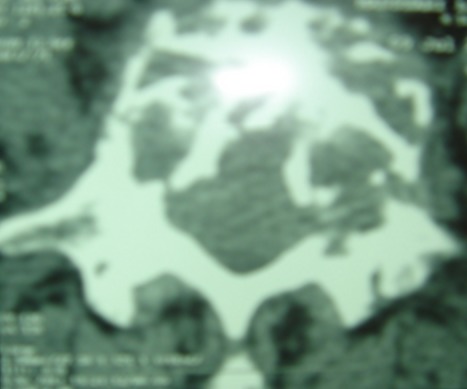
En coupe axiale: fracture tassement L5 avec recul du mur post, image en nid d'abeille

**Figure 3:**
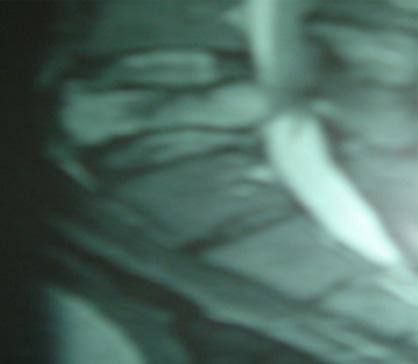
IRM en coupes sagittales T1,T2: fracture tassement de L5 vertèbre hyperintense qui se rehausse après injection de gadolinium

**Figure 4:**
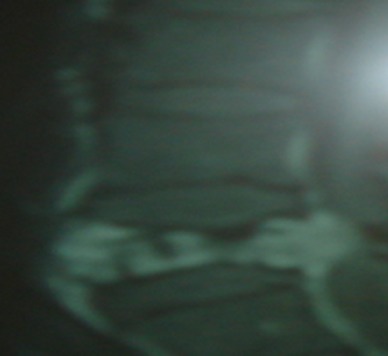
IRM en coupes sagittales T1,T2: fracture tassement de L5 vertèbre hyperintense qui se rehausse après injection de gadolinium

Le développement de l'hémangiome entraine une ostéolyse de l'os spongieux normal qui aboutit secondairement au développement réactionnel des travées verticales. En scanner par coupes axiales, ce développement aboutit au classique aspect en «nid d'abeille» (Figure 2). L'IRM occupe une place importante par le fait qu'elle permet une analyse multiplanaire directe et représente un pouvoir de caractérisation tissulaire. L'aspect habituel est caractérisé par un hyper signal osseux T1 et T2 (Figure 3, Figure 4). Elle trouve son intérêt majeur dans le bilan d'extension au niveau épidural avec une bonne approche du retentissement sur les structures nerveuses. Le traitement est difficile est aucune attitude thérapeutique ne fait l'unanimité. Il fait appel à une radiothérapie, une embolisation associée ou non à une chirurgie ou une vertebroplastie percutanée.

## Conclusion

L'hémangiome vertébral est une tumeur bénigne qui peut évoluée et provoquée des complications neurologiques graves, bien qu'aucun traitement ne soit nécessaire en présence des formes quiescentes, une surveillance clinique et radiologique est conseillée. Les formes douloureuses traduisent une évolutivité que la radiothérapie arrive généralement à enrayer. Les formes neurologiques nécessitent une prise en charge neurochirurgicale.
